# Measurement of Environmental Concern: A Review and Analysis

**DOI:** 10.3389/fpsyg.2020.00363

**Published:** 2020-03-06

**Authors:** Shannon M. Cruz, Brian Manata

**Affiliations:** Department of Communication Arts and Sciences, The Pennsylvania State University, University Park, PA, United States

**Keywords:** environmental attitudes, environmental concern, measurement, confirmatory factor analysis, environmental behavior

## Abstract

Growing concern about the seriousness of issues such as climate change has made the value of research on social and behavioral aspects of environmental problems clearer than ever. For authors studying environmental concern or attitudes, however, survey development can be a daunting task. A large number of scales measuring environmental concern have been developed, and it can be challenging to make informed decisions about which to use. To assist authors in navigating the literature, we present a review of existing scales, followed by two studies in which we examine the structural validity of five scales that are commonly implemented in this corpus and that adhere to classical test theory. These results have important implications for general issues with measurement in this area, and inform our recommendations for authors about key considerations when selecting and using environmental concern scales.

## Introduction

Understanding environmental attitudes is vital for addressing many applied environmental problems, ranging from local issues like water pollution to global issues like climate change. Effectively measuring environmental attitudes, however, is not always a simple task. Although today’s scholars have the good fortune of inheriting decades of measurement work from fields such as sociology, psychology, and education, navigating the vast number of scales available remains a challenge. Indeed, there is “an incredibly diverse set of measures or operational definitions of environmental concern” (Dunlap and Jones, 2002, p. 483; see also Heberlein, 1981; Klineberg et al., 1998), but a relative paucity of research to assess their validity and reliability.

In the interest of remedying this problem, this paper aims to provide a guide for those wishing to measure general environmental attitudes by (1) conducting a review of the instruments available, and (2) analyzing the quality of several of the most prominent instruments. This effort begins with a literature review assessing the state of environmental concern measures, follows with two studies designed to assess the psychometric properties of several prominent scales, and concludes with recommendations on the scales’ comparative utility in applied contexts.

## Background and Overview

In response to the growing environmental awareness observed in the 1970s, many scholars developed an interest in investigating environmental attitudes. As discussed at length by Dunlap and Jones (2002) and by others before them (e.g., Heberlein, 1981), although this flurry of activity provided a number of insights, it also created two major issues that remain largely unresolved. First, this shared interest often did not imply a shared view of how to conceptualize *environment* or *attitude*, leading to confusion and disagreement about issues of definition. Second, even those with a similar conceptualization of environmental attitudes often disagreed about how to measure attitudes effectively (see Dunlap, 2008, for a historical overview), leading to the creation of an incredibly large and diverse set of scales.

The focus of this paper is primarily on the second of these issues, but the first issue also bears discussing, as it provides the foundation of the approach taken in the rest of the paper. In doing so, the aim is not to resolve conceptual issues in the literature or to claim that one particular conceptualization is correct, but to articulate clearly how environmental attitudes are conceived of here for the purposes of the analyses that follow. This definition necessarily begins with a discussion of attitudes more generally.

### Defining Environmental Attitudes

For the purposes of this paper, two important assumptions are made about attitudes. First, we adopt a narrow definition of attitude, restricted to “the intensity of positive and negative affect toward concepts, persons, ideas, and other ‘objects’ in general” (Hunter et al., 1976, p. 3). Second, following in the psychological tradition that takes a structural approach to attitudes (Rokeach, 1968; Hunter et al., 1976), we assume that attitudes are connected to one another in a logical hierarchy. Higher-order attitudes are broad and abstract, and become progressively more specific and concrete as one moves down the hierarchy. Put together, these assumptions imply that (1) all specific attitudes about a topic are reflections of more general underlying attitudes; and (2) attitudes are distinct from values, beliefs, intentions, behavior, or other related concepts. In this paradigm, values are similar to attitudes, but are more general and abstract (Schwartz and Bilsky, 1987), such that one’s value system is connected to, but at a higher order than, one’s attitude system (Rokeach, 1968). Likewise, the attitude system is connected to, but distinct from, systems of beliefs, intentions, and behavior (Rokeach, 1968; Hunter et al., 1976).

This conceptualization of attitudes is certainly not the only one that has been identified, but it is the one most consistent with previous research demonstrating causal relationships between attitudes and behavior. In particular, the theory of reasoned action (TRA; Fishbein and Ajzen, 1975) states that attitudes and subjective norms predict behavioral intentions, which in turn predict behavior. There is overwhelming evidence in support of the theory, including multiple meta-analyses that corroborate the theory’s predictions (Sheppard et al., 1988; Kim and Hunter, 1993a, b) and document its ability to explain behavior in applied contexts, including condom use (Albarracín et al., 2001) and exercise (Hausenblas et al., 1997).

Environmental attitudes are not fundamentally different from other types of attitudes, and so can be defined and organized in the same way. In other words, one’s attitudes toward specific environmental topics are distinct in some ways, but are ultimately reflections of a single, broad environmental attitude—what is sometimes referred to as environmental concern (Dunlap and Jones, 2002). For example, one’s attitude toward a policy to protect the California condor might be one facet of a broader attitude toward endangered species protection, which may in turn be part of a broader attitude toward wildlife conservation, and so on. This view is consistent with the finding that recycling attitudes fit well into a hierarchy of views about resource availability (Padmanabhan, 1989) and with Heberlein 1981, p. 252) suggestion that the majority of environmental attitude scales “all measure some general orientation.” The distinction between environmental attitudes and environmental norms, intentions, and behaviors has also been demonstrated in previous research on the TRA, which finds the theory to have strong explanatory power for behaviors such as recycling (Goldenhar and Connell, 1993), climate change mitigation (Kim et al., 2012), and green consumption (Coleman et al., 2011). In sum, an environmental attitude can be defined both as *the intensity of positive or negative affect about a particular environmental topic* and as *a hierarchical attitude system that connects and organizes more specific attitudes about a range of environmental topics*.

There are a number of authors whose views of environmental attitudes are compatible with this one (e.g., Maloney et al., 1975; Stern and Dietz, 1994; Schultz, 2001), but many others who conceptualize them quite differently. For example, as Dunlap and Jones (2002) review, several authors consider beliefs, intentions, and behavior to be part of a single system, rather than as distinct systems. These differences of opinion are one reason why measurement of environmental concern has been so varied, though it is certainly not the only one. Setting aside conceptual differences, we turn now to issues of measurement within scales that target attitudes as defined in the preceding paragraphs.

### Issues in Measurement of Environmental Attitudes

To understand the measurement issues that have evolved in this subset of the literature, it is helpful to start with the early efforts to measure environmental attitudes in the 1970s. Several measures proliferated during this time, including Lounsbury and Tornatzky’s (1977) measure of attitudes toward environmental quality; Maloney and Ward’s (1973) measure of ecological attitudes and knowledge; Dunlap and Van Liere’s (1978) new environmental paradigm scale; and Weigel and Weigel’s (1978) environmental attitudes scale.

A number of criticisms were leveled against these early scales. For one, scholars pointed out several concerns about their face and content validity—the extent to which scales appeared valid on their face (Mosier, 1947; Blalock, 1972) and captured the pertinent aspects of environmental concern (Cronbach and Meehl, 1955; Kerlinger, 1964), respectively. Specifically, authors objected that scales were often atheoretical (Heberlein, 1981; Dunlap and Jones, 2002) and sometimes included items that were difficult for subjects to interpret (Arcury and Christianson, 1990; LaLonde and Jackson, 2002). The Maloney et al. (1975) and Weigel and Weigel (1978) scales were also dismissed as outdated with growing frequency as the years went on (see Bohlen et al., 1993; Dunlap et al., 2000; Dunlap and Jones, 2002; Milfont and Duckitt, 2010). As new environmental issues arose, it was suggested that these older scales no longer captured all relevant aspects of environmental concern. Issues were also raised about the scales’ structural validity (see Hunter and Gerbing, 1982). The underlying structures of these scales were often inconsistent with the measurement models proposed by the original authors (e.g., Smythe and Brook, 1980; see Dunlap, 2008), suggesting they were not effectively measuring what they purported to measure.

As objections to these classic scales mounted, it became abundantly clear that additional measurement work was needed. Unfortunately, the corresponding response was slower and more haphazard than one might have hoped. Although there were cases in which authors subsequently revised their scales in response to criticism (namely Dunlap et al., 2000), most instruments were never subjected to measurement work beyond their initial development. Instead, authors have continued to generate their own measures of environmental concern, including new, purportedly distinct scales (e.g., La Trobe and Acott, 2000) and *ad hoc* instruments for single studies (e.g., Vaske and Donnelly, 1999). This proliferation has resulted in an impressive number of available scales, most of which have received only limited attention and use.

## Review of Existing Scales

To examine the current state of measurement of environmental attitudes, it was necessary to compile a list of environmental attitude scales. To reiterate, we did not endeavor to compile a list of all environmental concern *measures*, only scales measuring environmental attitudes as distinct from beliefs, intentions, or behavior. Measures that draw on different conceptualizations of environmental attitudes, such as the Campbell paradigm (Kaiser et al., 2010, 2018), are outside of the focus of this paper.

Even setting these other types of measures aside, it was not possible to review every instrument, as there are hundreds of studies using idiosyncratic measures of environmental concern. Instead, scales were only included if they met three criteria. First, the scale had to appear in a published article. Scales developed for unpublished theses or dissertations (e.g., Adults’ Attitudes toward the Environment Scale, Malkus, 1992) were not included. Second, a primary goal of the article had to be scale development. This criterion eliminated instruments developed only for a single study (e.g., Buttel and Johnson, 1977; Guagnano and Markee, 1995; Vaske and Donnelly, 1999), without the intent to propose a scale for others’ use. Finally, the full set of items needed to be available in a published article. Not only was it more practical to include scales for which items were readily available, it was reasoned that authors would be unlikely to use scales for which items were unpublished (e.g., Maloney and Ward, 1973; McKechnie, 1977).

After reviewing 93 articles, 26 scales meeting these criteria were identified—18 measuring general environmental attitudes, five adapted for children, and two constructed for students (see Table 1). The scales included an average of 25.40 items (*SD* = 17.42) and purported to capture an average of 3.56 dimensions (*SD* = 2.69) of environmental concern. The first scales were developed in the 1970s (*n* = 5), and most others (*n* = 11) were published in the 1990s.

**TABLE 1:** Environmental attitude scales.

Scale Name	Author(s)	Citation Count	Items	Factors	Scale	Meas. Work
**General Scales**						
Socially Responsible Consumption Behavior Scale (SRCB)	Antil and Bennett, 1979	139	40	1	5	PCA
Ecological Worldview Scale (EWS)	Blaikie, 1992	104	24	7*	5	PCA
Ecological Concern Scale	Bohlen et al., 1993	245	15	4	5	EFA or PCA
Environmental Identity Scale	Clayton, 1993	(811)	24	1	2	EFA or PCA
New Environmental Paradigm Scale (NEP)	Dunlap and Van Liere, 1978	3365	12	1	4	PCA
Dominant Social Paradigm Scale (DSP)	Dunlap and Van Liere, 1984	653	29	8	4	PCA
New Ecological Paradigm Scale (Revised NEP)	Dunlap et al., 2000	4314	15	5*	5	PCA
Environmental Attitude Scale	Kuhn and Jackson, 1989	114	21	4		PCA
Modified NEP/DSP Environmental Attitudes Scale	La Trobe and Acott, 2000	183	21	4	4	EFA or PCA
Attitudes Toward Environmental Quality Scale	Lounsbury and Tornatzky, 1977	68	26	3	6	Cluster analysis
Ecological Attitudes and Knowledge Scale (Revised)	Maloney et al., 1975	684	45	4	2	None
Environmental Attitudes Inventory - Short Form (EAI-S)	Milfont and Duckitt, 2010	406	72	12*	7	CFA
Environmental Satisfaction Scale	Pelletier et al., 1996	54	8	2	7	EFA and CFA
Environmental Concerns	Schultz, 2001	1206	12	3	7	CFA
Value Orientations Scale	Stern et al., 1993	1962	9	3	4	None
Anthropocentric and Ecocentric Attitudes Scales	Thompson and Barton, 1994	1017	21	2	5	None
Environmental Concern Scale	Weigel and Weigel, 1978	586	16	1	4	None
Green Issues	Zimmer et al., 1994	402	57	7	27	PCA
**Scales for Children**						
Children’s Environmental Affect Scale (CEAS)	Erdogan and Marcinkowski, 2015	5	14	3	4	PCA
Children’s Environmental Attitude and Knowledge Scale (CHEAKS)	Leeming et al., 1995	352	66	2	5	EFA or PCA
New Ecological Paradigm Scale (Revised NEP) for Children	Manoli et al., 2007	186	10	5*	5	EFA and CFA
Children’s Attitudes Toward the Environment Scale – Preschool Version (CATES-PV)	Musser and Diamond, 1999	156	15	1	2	None
Children’s Attitudes Toward the Environment Scale (CATES)	Musser and Malkus, 1994	168	25	1	2	None
**Scales for Student Groups**						
Environmental Attitude Scale	Berberoglu and Tosunoglu, 1995	109	18	4	5	PCA and CFA
Environmental Attitudes of the University Scale	Fernández-Manzanal et al., 2007	120	20	1	5	EFA and CFA

Citation counts were taken from Google Scholar (scholar.google.com) on June 27, 2018; for the Clayton (1993) source, the citation count pertains to the book in which this chapter was published. The *Items* column refers to the number of items retained in the final version of the scale reported by the authors. The *Factors* column refers to the number of factors produced or proposed in the final version of the scale reported by the authors; if no factor structure was specified, a unidimensional solution was assumed; the * indicates a second-order factor structure was also found. The *Scale* column refers to the number of scale points used for most or all of the scale factors; note that some subscales (e.g., the knowledge subscale from Maloney et al., 1975) use a different number of scale points from the number provided. The *Meas. Work* column refers to the structural validity work performed by the authors when proposing the scale; CFA, confirmatory factor analysis; EFA, exploratory factor analysis; PCA, principal components analysis; ‘EFA or PCA’ indicates that the method was exploratory, but not further specified by the authors.

As indicated by the citation counts, a few scales stand out as particularly popular (Stern et al., 1993; Dunlap et al., 2000; Schultz, 2001), and most others have received at least modest attention since their creation. However, examining the literature also revealed that citations do not necessarily imply actual scale use. Indeed, five of the scale papers listed in Table 1 alone cited the Maloney et al. (1975) scale (Antil and Bennett, 1979; Bohlen et al., 1993; Leeming et al., 1995; Milfont and Duckitt, 2010), but only one (Musser and Diamond, 1999) actually made use of the scale itself. Moreover, even in cases where established scales are used, they are often modified beforehand, such that any associated measurement work does little to inform readers as to the quality of the original scales. Hawcroft and Milfont 2010, p. 143) have documented this kind of “use (and abuse)” among studies using the new ecological paradigm scale (NEP, Dunlap et al., 2000), and other scales have been subjected to similar treatment (e.g., see Dispoto, 1977; Schahn and Holzer, 1990).

What this review also makes clear is that the initial measurement work used in developing most of these scales was inappropriate or inadequate. Several (*n* = 6) were proposed without any examination of structural validity, and most others were assessed using exploratory factor analysis (EFA; *n* = 3) or principal components analysis (PCA; *n* = 9), sometimes without clarifying whether EFA or PCA was being employed (*n* = 4). In contrast, only a few scales (*n* = 6) were examined using confirmatory factor analysis (CFA; see Hunter and Gerbing, 1982).

These practices are troubling for at least two reasons. First, relying only on face and content validity can be misleading (Mosier, 1947). Even though a scale may look, on its face, to be a strong and coherent instrument, it may turn out to be a poor representation of the data. Second, neither EFA nor PCA is well suited for testing the fit of established scales to a specified measurement model (Hunter and Gerbing, 1982; Park et al., 2002)—alternatively, CFA is designed to test hypotheses regarding the latent factor structure underlying a set of items. Although there are some cases where CFA may be inappropriate, such as when non-linear item-total relationships are expected (e.g., Guttman or Rasch models; see Keating and Boster, 2019) or when one-item measures are used, these features are not evident in the scales reviewed here.

As such, a clear step forward would be to conduct additional measurement work on these scales by making use of CFA. For some, this would be the first time structural validity has been tested empirically. For others that were tested with CFA to begin with and for the NEP, which has subsequently been subjected to CFA by other authors (e.g., see Xiao and Dunlap, 2007; Amburgey and Thoman, 2012; Xiao et al., 2013), further CFA work would address questions of replication and scale invariance (see Levine et al., 2006). To this end, two studies were conducted to contribute to general understanding of measurement in this area, as well as to examine the relative utility of the available scales. A decision was made to focus only on the general scales (*n* = 18) for this analysis, as these would be presumably useful to the widest range of scholars. Given that it was not possible to examine all of these scales, several criteria were used to narrow down this list to a more manageable set of instruments for analysis. First, when scales used some of the same items (and were thus redundant), all but one was eliminated from consideration (ruling out Dunlap and Van Liere, 1978; Antil and Bennett, 1979; Kuhn and Jackson, 1989; Blaikie, 1992; Milfont and Duckitt, 2010). Second, when multiple scales were based on the same theoretical foundation, all but one scale was again eliminated from consideration, in order to reduce overlap (ruling out Dunlap and Van Liere, 1984; Bohlen et al., 1993; Stern et al., 1993; Thompson and Barton, 1994; La Trobe and Acott, 2000). Of the remaining scales (Maloney et al., 1975; Lounsbury and Tornatzky, 1977; Weigel and Weigel, 1978; Clayton, 1993; Zimmer et al., 1994; Pelletier et al., 1996; Dunlap et al., 2000; Schultz, 2001), preference was given to the more popular and classic scales.

## Study 1

We begin this investigation with Weigel and Weigel’s (1978) environmental concern scale (EC), a classic scale which has received a substantial number of citations, but has also been classified as outdated by several authors (Dunlap et al., 2000; Dunlap and Jones, 2002; Milfont and Duckitt, 2010). The NEP (Dunlap et al., 2000) offers an excellent standard to which the EC scale can be compared, both because it is more recently updated and because Dunlap et al. (2000) explained the popularity of the NEP as resulting in part because of the “dated” nature of scales such as the EC (p. 427).

### Method

#### Sample

Data were collected using Amazon’s Mechanical Turk (mTurk), a crowd-sourcing platform that allows companies or researchers to pay workers to complete Human Intelligence Tasks or HITs (Amazon Mechanical Turk, 2020). Four hundred workers were requested, and each participant received $0.05 for completing the survey.

#### Measures

The survey included the 15-item revised NEP scale and the 16-item EC scale. The revised NEP scale is proposed to fit a five-factor solution, tapping into five distinct aspects of environmental concern: *limits to growth* (e.g., “We are approaching the limit of the number of people the earth can support”), *anti-anthropocentrism* (e.g., “Humans have the right to modify the natural environment to suit their needs”), *fragility of nature’s balance* (e.g., “When humans interfere with nature it often produces disastrous consequences”), *rejection of exemptionalism* (e.g., “Human ingenuity will insure that we do NOT make the earth unlivable”), and *possibility of an eco-crisis* (e.g., “Humans are severely abusing the environment”). Each subscale is composed of three items.

The EC scale is proposed to fit a unidimensional solution, with all 16 items reflecting general environmental concern. All items were measured on five-point Likert-type scales (1 = *strongly disagree*, 5 = *strongly agree*).

#### Analysis Procedure

CFA was used to assess the extent to which the NEP and EC scales fit their proposed measurement models. Data were analyzed utilizing the lessR package (Gerbing, 2014) of the R 3.1.0 statistical software (R Core Development Team, 2014), which employs the centroid solution to estimate parameters (Hunter and Gerbing, 1982).

Structural validity was examined in two stages: (1) first-order CFA, to examine the dimensionality of the items in each scale; and (2) second-order CFA, to examine whether or not constructs from both scales were indicators of a higher-order latent factor (see Hunter and Gerbing, 1982). The first stage served the purpose of evaluating a scale’s structure (how many specific attitudes are measured) and quality (how well the proposed indicators capture each one). To do so, obtained correlations between items were compared to the correlations predicted by the internal consistency and parallelism theorems. The internal consistency theorem specifies that the correlation between two indicators of the same factor (*x*_*i*_ and *x*_*j*_) will be equal to the product of the correlations of each indicator with the factor true score (*T*), which are estimated as their factor loadings:

$$r_{x_{i}\,x_{j}}=r_{x_{i}\,T}\,r_{x_{j}\,T}$$

The parallelism theorem specifies that the correlation between two indicators of different factors (*x*_*i*_ and *y*_*k*_) will be equal to the product of the correlations between each indicator with its respective factor true score (*T* or *U*) and the correlation between the two true scores:

$$r_{x_{i}\,y_{k}}=r_{x_{i}\,T}\,r_{T\,U}\,r_{U\,y_{k}}$$

The larger and more numerous the deviations between observed scores and the scores predicted by these theorems, the poorer the model fit. The fit of the model was assessed using the root mean square error (RMSE), comparative fit index (CFI), and Akaike information criterion (AIC).^1^ Although cutoff values for the RMSE are not well established, lower values represent smaller errors on aggregate and thus better model fit (see Hunter and Gerbing, 1982). Hu and Bentler 1999, p. 27) recommend a cutoff value of or close to 0.95 for the CFI, and, although there are no recommended cutoffs for the AIC, lower AIC values (among nested models) indicate superior fit (Singer and Willet, 2003).

If model fit was poor, the correlation matrices and R outputs were examined with the intent of improving the fit of each scale. In cases where the factor structure appeared to be misspecified, improvement involved specifying an alternate structure that better reflected the underlying factors. In cases where there were invalid items (i.e. items with unacceptably large residuals, significant at *p* < 0.05; see Hunter and Gerbing, 1982), improvement involved removing them from their respective factors.^2^

Once good fit was obtained for each scale, the factors were examined using second-order CFA. The logic of this analysis is the same as the first-order CFA, but is concerned with the unidimensionality of a set of factors (i.e. second-order unidimensionality) rather than a set of items. This analysis permitted investigation of the extent to which the different factors of the NEP and EC held together both within and across the two scales. In other words, this stage tested the fit of the different environmental attitudes to a hierarchical structure.

### Results

#### NEP Scale

Unexpectedly, the first-order CFA of the NEP scale revealed that Dunlap et al. (2000) proposed five-factor structure produced very poor model fit (RMSE = 0.20, CFI = 0.59, AIC = 865.21). The RMSE was high, and the CFI fell well below accepted standards. Moreover, reliability coefficients were low across the five factors (αs = 0.39–0.59; ωs = 0.45–0.75).

To investigate causes of this structural invalidity, patterns of relationships in the item correlation matrix were examined in more detail. This perusal suggested that the items might be better reflected by a three-factor solution, with factors addressing *limits to growth*, *anti-anthropocentrism*, and *concern about ecological damage*. Similar three-factor structures have been uncovered by other authors (e.g., Albrecht et al., 1982), so proceeding with this alternative model was not unprecedented. Items were repositioned accordingly, and then a follow-up CFA was conducted to assess the fit of this alternative three-factor solution.

After removing a few items associated with exceedingly large residuals, the resulting model provided decidedly better fit to the data (RMSE = 0.05, CFI = 0.96, AIC = 159.89), and reliability coefficients also evidenced substantial increases (αs = 0.68–0.80; ωs = 0.69–0.80). As a result, this alternative model was retained for comparison with the EC scale (see Table 2 for the final factor structure).

**TABLE 2:** Final results of confirmatory factor analysis for the revised new ecological paradigm scale (Study 1: *N* = 399, Study 2: *N* = 326).

	Study 1					Study 2				
		
	F1	F2	F3	*M*	*SD*	F1	F2	F3	*M*	*SD*
***Limits to growth* (α = 0.70, 0.71; ω = 0.70, 0.71)**										
We are approaching the limit of the number of people the earth can support. [NEP 1]	0.73			3.54	1.05	0.74			3.33	1.19
The earth is like a spaceship with very limited room and resources. [NEP 11]	0.73			3.47	1.14	0.74			3.67	1.13
***Anti-anthropocentrism* (α = 0.80, 0.80; ω = 0.80, 0.81)**										
* Humans have the right to modify nature to suit their needs. [NEP 2]		0.69		2.97	1.24		0.65		3.37	1.12
* Human ingenuity will ensure that we do NOT make the earth unlivable. [NEP 4]		0.53		2.70	1.03		0.49		3.16	1.10
* The balance of nature is strong enough to cope with the impacts of modern industrial nations. [NEP 8]		0.73		2.84	1.17		0.78		3.63	1.15
* The so-called ecological crisis facing humankind has been greatly exaggerated. [NEP 10]		0.74		2.88	1.22		0.83		3.65	1.27
* Humans were meant to rule over the rest of nature. [NEP 12]		0.63		2.97	1.28		0.59		3.35	1.35
***Concern about ecological damage* (α = 0.68,0.77; ω = 0.69,0.79)**										
When humans interfere with nature it often produces disastrous consequences. [NEP 3]			0.55	3.81	0.98			0.61	3.83	0.96
Humans are severely abusing the environment. [NEP 5]			0.74	3.94	0.92			0.81	3.97	1.01
Despite our special abilities, humans are still subject to the laws of nature. [NEP 9]			0.38	3.98	0.86			0.47	4.26	0.79
If things continue on their present course, we will soon experience a major ecological catastrophe. [NEP 15]			0.71	3.91	0.99			0.84	3.68	1.12

Reverse coded items are indicated with an asterisk (*). These items were recoded before calculating means.

#### EC Scale

Similar to the NEP, inspection of the residual matrix indicated that the unidimensional solution proposed by Weigel and Weigel (1978) produced poor fit (RMSE = 0.22, CFI = 0.57, AIC = 1330.14). The scale was reliable by conventional standards (α = 0.83; ω = 0.89), but these coefficients are likely inflated by the scale’s large number of items (see Nunnally et al., 1967). Moreover, adequate reliability does not imply valid measurement (Hunter and Gerbing, 1982; Levine, 2005). As such, item content and residuals were evaluated with the intent of improving model fit.

Similar to Dunlap et al. (2000) scale, examining the patterns in the correlation matrix suggested that an alternate two-factor structure would improve model fit. Based on the item content, the two factors were labeled *concern about pollution* (e.g., “The federal government will have to introduce harsh measures to halt pollution since few people will regulate themselves”) and *rejection of industrial status quo* (e.g., “Industry is doing its best to develop effective anti-pollution technology”). The items were repositioned accordingly, and a follow-up CFA was performed on the modified factor structure.

After removing a few items that evidenced exceedingly large residuals, the new model produced markedly better fit to the data (RMSE = 0.04, CFI = 0.98, AIC = 82.35). Moreover, although reliability was attenuated (αs = 0.70, 0.79; ωs = 0.71, 0.79), the coefficients remained acceptable (Nunnally et al., 1967). Therefore, the two-factor solution was preferred to Weigel and Weigel’s (1978) proposed unidimensional measurement model (see Table 3).

**TABLE 3:** Final results of confirmatory factor analysis for the Weigel and Weigel scale (Study 1: *N* = 390, Study 2: *N* = 325).

	Study 1				Study 2			
		
	F1	F2	*M*	*SD*	F1	F2	*M*	*SD*
***Concern about pollution* (α = 0.70, 0.74; ω = 0.79, 0.74)**								
The federal government will have to introduce harsh measures to halt pollution since few people will regulate themselves. [EC 1]	0.72		3.75	0.97	0.66		3.56	1.16
I’d be willing to make personal sacrifices for the sake of slowing down pollution even though the immediate results may not seem significant. [EC 3]	0.58		3.76	0.91	0.65		3.78	0.92
The government should provide each citizen with a list of agencies and organizations to which citizens could report grievances concerning pollution. [EC 10]	0.69		3.80	0.98	0.79		3.70	1.02
***Rejection of industrial status quo* (α = 0.71, 0.69; ω = 0.79, 0.70)**								
* Although there is continual contamination of our lakes, streams, and air, nature’s purifying processes soon return them to normal. [EC 8]		0.77	3.00	1.19		0.48	4.01	1.00
* Predators such as hawks, crows, skunks, and coyotes which prey on farmers’ grain crops and poultry should be eliminated. [EC 11]		0.76	3.31	1.29		0.68	4.15	0.94
* The currently active anti-pollution organizations are really more interested in disrupting society than they are in fighting pollution. [EC 12]		0.60	2.74	1.07		0.52	3.44	1.17
* Even if public transportation was more efficient than it is, I would prefer to drive my car to work. [EC 13]		0.51	2.80	1.13		0.59	3.11	1.21
* Industry is doing its best to develop effective anti-pollution technology. [EC 14]		0.64	2.74	1.07		0.53	3.34	1.06

Reverse coded items are indicated with an asterisk (*). These items were recoded before calculating means.

#### Second-Order Structure

After establishing a valid factor structure for both the NEP and the EC scale, the analysis proceeded with a second-order CFA. Inspection of the residual matrix revealed a decided lack of internal consistency^3^ among the five factors (RMSE = 0.25, CFI = 0.47, AIC = 427.76). Closer inspection of the residual matrix, however, indicated that a disproportionate number of errors were attributed to the EC scale’s second factor, *rejection of industrial status quo*. When this factor was removed, the results indicated excellent model fit (RMSE = 0.05, CFI = 0.98, AIC = 31.46). This finding reveals that the three NEP factors and the *concern about pollution* factor from the EC are not distinct measures; they are all indicators of the same latent environmental concern construct (see Table 4).

**TABLE 4:** Study 1: second-order confirmatory factor analysis.

	F1	*M*	*SD*
Limits to growth [NEP]	0.46	3.51	0.96
Anti-anthropocentrism [NEP]	0.20	2.87	0.89
Concern about ecological damage [NEP]	0.90	3.91	0.67
Concern about pollution [EC]	0.80	3.77	0.76
Rejection of industrial *status quo* [EC]	–	2.92	0.85

*Bracketed abbreviations indicate the scale of which that factor is a part. NEP, new ecological paradigm scale; EC, Weigel and Weigel scale. Factor loadings are not provided (–) for factors that were dropped from the second-order model.*

### Discussion

Study 1 assessed the validity of both Dunlap et al. (2000) revised NEP scale and Weigel and Weigel’s (1978) EC scale. In general, both proposed factor structures failed to produce good fit. Fit statistics were poor, and the number of unacceptably large residual terms was high. To resolve these issues, the patterns of relationships evident in the residual and correlation matrices were inspected in greater detail. This procedure uncovered a structurally valid solution for both the NEP and EC. Specifically, instead of Dunlap et al.’s (2000) five-factor solution for the NEP, a three-factor model, with some items excluded, provided better fit to the data. Interestingly, the factors resembled those found in other measurement studies, several of which have identified limits to growth, anti-anthropocentrism, and the balance of nature as key themes (e.g., Albrecht et al., 1982; see also Dunlap, (2008). Likewise, inspection of the EC scale revealed that the data were better represented by a two-factor structure, with some invalid items removed, rather than the one-factor solution proposed by Weigel and Weigel (1978). Examination of the item groupings in the final solution also suggested a coherent structure; one factor appeared to tap into subjects’ concern about harmful effects of pollution, and the other appeared to measure perceptions of harmful industrial practices.

Subsequent tests of the second-order unidimensional model further revealed that all three NEP factors and one EC factor are driven by the same latent construct (environmental concern). Thus, although each of the four factors measure different aspects of environmental concern, they all reflect the same higher-order attitude. All four factors may thus be considered general measures of environmental concern. Conversely, these data suggest that the EC scale’s *rejection of industrial status quo* factor is distinct from the others. Rather than indicating general environmental concern, this scale appears to measure attitudes toward harmful industrial practices. Presumably, measures of environmental concern will be strongly correlated with this unique factor, but the two types of measures are not interchangeable.

Despite these contributions, there are two limitations that merit discussion. First, data collection for Study 1 was not limited to any particular sample, and the multinational nature of typical mTurk samples (Ross et al., (2010) could conceivably impact the validity of the solutions produced here (e.g., Inglehart, 1995). Second, the three- and two-factor solutions produced in Study 1 were determined in a somewhat exploratory fashion. Although we do not see removal of items as an exploratory practice, we agree that modifying the factor structure of a scale constitutes one. In other words, additional work is needed to reveal whether these factor structures can be substantiated by additional data or are merely artifacts of chance (Anderson and Gerbing, 1988). Although this issue is less concerning for the NEP scale, given that similar three-factor solutions have been found in the past, there is no previous work to corroborate the alternate two-factor solution identified for the EC scale.

## Study 2

To allay the limitations of Study 1, a second study was conducted in which the NEP and EC scales were once again investigated. Examining these scales a second time permitted the opportunity to confirm and replicate the alternative factor structures identified in Study 1. Study 2 also held nationality constant by collecting data from U.S. residents only.

Three additional scales were also examined in this study: two classic scales (Lounsbury and Tornatzky, 1977, or LT; and Maloney et al., 1975, or MWB) and one popular, more modern scale (Schultz, 2001, or SC). Similar to the first study, the five scales were examined for first-order structural validity, and then explored using second-order CFA.

### Method

#### Sample

Data were collected using Amazon’s mTurk website. The sample included *N* = 326 workers, and the data collection was restricted such that only U.S. residents could participate. This stipulation removed any concern that the factor structure obtained in the first study was an artifact of a multinational sample. Each worker was rewarded $0.10 for completing the survey.

The sample was predominantly female (58.0%) and White (81.6%), and tended to be younger (*M* = 37.27 years, *SD* = 13.27). Most participants identified as Democrats (40.8%) or Independents (23.9%), and also tended to be politically liberal (49.4% somewhat or strongly liberal). The sample also tended to be well educated, with most participants having either some college experience (37.4%) or a Bachelor’s degree (27.3%).

#### Measures

The survey included the 15-item revised NEP scale, 16-item EC scale, 12-item LT scale, 30-item MWB scale, and 12-item SC scale. As described in Study 1, the NEP was originally proposed to fit a five-factor solution, but was found to fit an alternative three-factor solution; the EC scale was originally proposed to fit a unidimensional solution, but was found to fit an alternative two-factor solution. The Lounsbury and Tornatzky (1977) scale was proposed to fit a three-factor solution, with subscales measuring *concern for environmental degradation* (five items; e.g., “If mankind is going to survive at all, environmental pollution must be stopped”), *concern for environmental action* (five items; e.g., “People should buy (and return) beverages only in returnable containers”), and *concern for overpopulation* (two items; e.g., “Every couple in America should try not to have more than two children”). The Maloney et al. (1975) scale was proposed to fit a three-factor solution^4^, with subscales reflecting *affect* (10 items; e.g., “1 feel people worry too much about pesticides on food products”)—constituting the only attitude scale for the purposes of the present paper—*verbal commitment* (10 items; e.g., “I’d be willing to ride a bicycle or take the bus to work in order to reduce air pollution”), and *actual commitment* (10 items; e.g., “I subscribe to ecological publications”). Finally, the Schultz (2001) scale was also proposed to fit a three-factor solution, with subscales measuring *biospheric concern* (four items; e.g., “I am concerned about environmental problems because of the consequences for birds”), *egoistic concern* (four items; e.g., “I am concerned about environmental problems because of the consequences for me”), and *social-altruistic concern* (four items; e.g., “I am concerned about environmental problems because of the consequences for all people”).

For the NEP, EC, LT, and SC scales, as well as the verbal commitment and affect factors of the MWB scale, the items were measured on five-point Likert-type scales (1 = *strongly disagree*, *5* = *strongly agree*). For the actual commitment factor of the Maloney et al. (1975) scale, the items were also measured on five-point Likert-type scales, but with different scale points (1 = *never*, 5 = *regularly*).^5^

#### Analysis Procedure

For testing the revised NEP and EC scales, both the original structure and the revised structure were reexamined and compared. For the SC scale, LT scale, and MWB scale, the authors’ predicted three-factor models were examined. Analytic procedures remained the same as for Study 1.

### Results

#### NEP Scale

Although still worse than desired, model fit for Dunlap et al. (2000) proposed five-factor solution was superior to the fit in Study 1. Specifically, fit statistics improved noticeably (RMSE = 0.08, CFI = 0.93, AIC = 334.78). Moreover, although some of the reliability coefficients were lower than desired, many were acceptable by conventional standards (αs = 0.60–0.86; ωs = 0.63–0.87). Nevertheless, model fit improved markedly when the alternative three-factor solution produced in Study 1 was employed. The analysis indicated that model fit was superior (RMSE = 0.05, CFI = 0.96, AIC = 172.16), and reliability coefficients improved as well (αs = 0.71–0.80; ωs = 0.71–0.81). As a result, the three-factor structure was again retained for comparison with the three classic scales (see Table 2).

#### EC Scale

Similar to the NEP scale, the fit of Weigel and Weigel’s (1978) one-factor solution in this study was better than in Study 1. Fit statistics evidenced noticeable improvements (RMSE = 0.07, CFI = 0.85, AIC = 478.79) and reliability coefficients remained high (α = 0.90; ω = 0.90). Nevertheless, model fit improved when the data were tested using the alternative two-factor solution uncovered in Study 1 (RMSE = 0.06, CFI = 0.96, AIC = 93.69). Reliability coefficients also remained adequate, although they were somewhat lower (αs = 0.69, 0.74; ωs = 0.70, 0.74). The two-factor solution thus produced superior model fit across samples and studies (see Table 3).

#### SC Scale

Schultz’s (2001) proposed three-factor solution fared reasonably well (RMSE = 0.06, CFI = 0.94, AIC = 333.94) and reliabilities were exceptionally high (αs = 0.89–0.95; ωs = 0.90–0.95). Nevertheless, inspection of the residual matrix indicated that several items were associated with large residuals, suggesting fit of the model could be improved further. Indeed, removal of problematic items produced better fit to the data (RMSE = 0.03, CFI = 0.96, AIC = 172.67) without reducing reliability (αs = 0.91–0.95; ωs = 0.91–0.95). Hence, although Schultz’s initially proposed three-factor solution evidenced adequate fit, model fit could be further improved with the removal of a few invalid items (see Table 5).

**TABLE 5:** Study 2: final results of confirmatory factor analysis for the Schultz scale (*N* = 313).

	F1	F2	F3	*M*	*SD*
***Biospheric concern* (α = 0.95, ω = 0.95)**					
Plants [SC 1]	0.86			4.03	1.04
Marine life [SC 2]	0.94			4.15	0.97
Birds [SC 3]	0.91			4.07	0.94
Animals [SC 4]	0.91			4.25	0.84
***Egoistic concern* (α = 0.92, ω = 0.92)**					
Me [SC 5]		0.81		4.28	0.95
My health [SC 7]		0.91		4.32	0.92
My future [SC 8]		0.93		4.19	1.01
***Social-altruistic concern* (α = 0.91, ω = 0.91)**					
All people [SC 10]			0.91	4.26	0.94
Children [SC 11]			0.91	4.38	0.89

*The prompt for each item was “I am concerned about environmental problems because of the consequences for_____________.*

#### LT Scale

The three-factor solution proposed by Lounsbury and Tornatzky (1977) evidenced marginal fit (RMSE = 0.07, CFI = 0.89, AIC = 263.99), but the CFI was lower than conventional cutoffs. Moreover, although reliability coefficients were acceptable for the *concern for environmental degradation* (α = 0.83; ω = 0.83) and *concern for environmental action* factors (α = 0.70; ω = 0.71), reliability coefficients for the *concern for overpopulation* factor were lower than might be desired (α = 0.63; ω = 0.63).

To improve model fit, two items that produced substantial errors were removed from the measurement model (see Table 6). Removal of these items produced comparatively better fit (RMSE = 0.06, CFI = 0.95, AIC = 141.33), but lowered the reliability of the *concern for environmental action* factor appreciably (α = 0.62; ω = 0.64). Thus, although evidence for the predicted three-factor solution was favorable, development of additional items would help improve the low reliabilities of some of the factors (Nunnally et al., 1967).

**TABLE 6:** Study 2: final results of confirmatory factor analysis for the Lounsbury and Tornatzky scale (*N* = 325).

	F1	F2	F3	*M*	*SD*
***Concern for environmental degradation* (α = 0.82, ω = 0.82)**					
* The news media have exaggerated the ecological problem. [LT 1]	0.74			3.58	1.21
If mankind is going to survive at all, environmental pollution must be stopped. [LT2]	0.74			3.75	1.07
I am worried about future children’s chance of living in a clean environment. [LT 4]	0.71			3.86	1.04
* We shouldn’t worry about environmental problems because science and technology will solve them before very long. [LT 5]	0.70			3.97	1.00
***Concern for environmental action* (α = 0.62, ω = 0.64)**					
People should buy (and return) beverages only in returnable containers. [LT 6]		0.52		3.67	0.94
People should use less detergent than the manufacturer recommends to help preserve water quality. [LT 7]		0.75		3.34	0.99
* There is nothing wrong with using electric can openers, electric pencil sharpeners, and electric toothbrushes. [LT 8]		0.57		2.76	1.06
* Putting a brick in one’s toilet to conserve water is a dumb idea.		0.34		3.13	1.28
***Concern for overpopulation* (α = 0.63, ω = 0.63)**					
Every couple in America should try not to have more than two children. [LT 11]			0.68	2.85	1.28
Overpopulation is a major source of environmental problems today. [LT 12]			0.68	3.52	1.13

Reverse coded items are indicated with an asterisk (*). These items were recoded before calculating means.

#### MWB Scale

The initial CFA indicated that Maloney et al. (1975) proposed three-factor solution produced poor model fit (RMSE = 0.12, CFI = 0.74, AIC = 1913.14). Moreover, although reliability coefficients were high (αs = 0.85–0.91; ωs = 0.85–0.91), these coefficients are likely inflated by the large number of items (10) assigned to each of the three factors.

In an attempt to improve model fit, numerous problematic items were removed. This procedure produced markedly better model fit (RMSE = 0.05, CFI = 0.97, AIC = 197.03), and reliability coefficients remained acceptable (αs = 0.73–0.88; ωs = 0.74–0.88). The MWB scale thus provided acceptable fit to the data when several problematic items were removed (see Table 7).

**TABLE 7:** Study 2: final results of confirmatory factor analysis for the Maloney et al. (1975) scale (*N* = 324).

	F1	F2	F3	*M*	*SD*
***Verbal commitment* (α = 0.73, ω = 0.74)**					
I’d be willing to ride a bike or take the bus to work in order to reduce air pollution. [MWB 1]	0.67			3.50	1.17
* I would probably never join a group or club which is concerned solely with ecological issues [MWB 2]	0.68			3.11	1.19
* I’m not willing to give up driving on a weekend due to a smog alert. [MWB 4]	0.59			3.29	1.11
I would donate a day’s pay to a foundation to help improve the environment. [MWB 6]	0.63			2.77	1.16
***Actual commitment* (α = 0.88, ω = 0.88)**					
I have written to a congressmen concerning pollution problems. [MWB 13]		0.80		1.64	1.11
I have contacted community agencies to find out what I can do about pollution. [MWB 14]		0.80		1.79	1.06
I have attended a meeting of an organization specifically concerned with bettering the environment. [MWB 16]		0.85		1.80	1.13
I have joined cleanup drives. [MWB 18]		0.74		2.13	1.18
***Affect* (α = 0.83, ω = 0.84)**					
It genuinely infuriates me to think that the government doesn’t do more to help control pollution of the environment. [MWB 23]			0.76	3.53	1.15
I become incensed when I think about the harm being done to plant and animal life by pollution. [MWB 25]			0.80	3.54	1.06
* I’m usually not bothered by so-called “noise pollution.” [MWB 26]			0.44	3.38	1.17
When I think of the ways industries are polluting, I get frustrated and angry. [MWB 28]			0.79	3.67	1.02
* The whole pollution issue has never upset me too much since I feel it’s somewhat overrated. [MWB 29]			0.74	3.70	1.14

Reverse coded items are indicated with an asterisk (*). These items were recoded before calculating means.

#### Second-Order Factor Structure

Similar to Study 1, once structural validity had been established for each of the five scales, they were examined further using second-order CFA. As before, if each of these scales reflects a higher-order environmental concern factor, then the 14 factors would be expected to fit a second-order unidimensional model. However, there were two qualifiers to this prediction. First, Study 1 revealed that the EC’s *rejection of industrial status quo* factor did not fit with the second-order model. Therefore, we anticipated that it might cause problems in this model as well. Second, the SC was proposed specifically to tap into different value systems—concern about environmental consequences for oneself (*egoistic concern*), for humans in general (*social-altruistic concern*), and for the environment (*biospheric concern*). Whereas *biospheric concern* clearly seems to tap into environmental concern, the other factors may not necessarily do so. As a result, it was expected that *egoistic concern* and *social-altruistic concern* might also cause problems in the second-order model. Finally, it was expected that if the MWB’s *verbal commitment* and *actual commitment* subscales truly evaluate intentions and behavior, respectively, they should be distinct from the *affect* subscale and other attitude scales.

A solution in which all factors were predicted by the same latent factor (environmental concern) provided a poor fit to the data (RMSE = 0.09, CFI = 0.83, AIC = 723.61). Thus the hypothesis that all measured scales tap the same underlying construct of environmental concern is not supported. Again, given the results of the first study, this was not entirely inconsistent with expectations. Inspection of the residual matrix confirmed that the same EC scale factor was once again problematic in this analysis, and also substantiated our suspicions that the SC scale’s *egoistic concern* and *social-altruistic concern* tapped into constructs other than environmental concern. The MWB scale’s *actual commitment* factor also failed to fit with the others, though this was not the case for *verbal commitment*. The LT scale’s *concern for overpopulation* factor also turned out to be problematic (see Table 8).

**TABLE 8:** Study 2: second-order confirmatory factor analysis.

	F1	*M*	*SD*
Limits to growth [NEP]	0.72	3.50	1.02
Anti-anthropocentrism [NEP]	0.83	3.43	0.89
Concern about ecological damage [NEP]	0.86	3.93	0.76
Concern about pollution [EC]	0.83	3.68	0.084
Rejection of industrial *status quo* [EC]	–	3.61	0.72
Concern for environmental degradation [LT]	0.91	3.79	0.87
Concern for environmental action [LT]	0.72	3.23	0.073
Concern for overpopulation [LT]	–	3.18	1.03
Biospheric concern [SC]	0.64	4.12	0.88
Egoistic concern [SC]	–	4.26	0.89
Social-altruistic concern [S]	–	4.31	0.87
Verbal commitment [MWB]	0.71	3.17	0.86
Actual commitment [MWB]	–	1.82	0.95
Affect [MWB]	0.90	3.56	0.85

*Bracketed abbreviations indicate the scale of which that factor is a part. NEP, new ecological paradigm scale; EC, Weigel and Weigel scale; LT, Lounsbury and Torntazky scale; SC, Schultz scale; MWB, Maloney et al. (1975) scale. Factor loadings are not provided (–) for factors that were dropped from the second-order model.*

Once these factors were removed from the second-order model, model fit improved markedly (RMSE = 0.03, CFI = 0.96, AIC = 163.73), with all three fit indices evidencing substantial improvements. Thus, it may be concluded that a majority of factors investigated in this study are driven by the same underlying environmental concern construct. The remaining factors, on the other hand, cannot be considered measures of general environmental concern.

## General Discussion

The purpose of these two studies was to examine the structural validity of five available measures of environmental concern. The scales were tested separately using first-order CFA, and then a higher-order factor structure was explored using second-order CFA. Across the two studies, it was possible to establish valid factor structures for all five scales. Regardless of whether the final factor structure was as the authors originally proposed (as was the case for the LT, MWB, and SC scales) or a modified version thereof (as was the case for the EC and NEP scales), good first-order fit was ultimately obtained for each scale. The second-order CFA also revealed remarkable overlap among the scales, although a few specific factors were found not to be valid indicators of environmental concern.

These results have a few important implications for measurement in this area. First, they suggest that claims about the outdatedness and invalidity of classic measures of environmental concern are largely unsubstantiated. The three classic scales examined here all exhibit structural validity and incorporate at least one factor that is second-order unidimensional with other measures of environmental concern. Furthermore, the findings suggest that although the more recently developed scales also perform well psychometrically, they are not necessarily superior to older scales. The first- and second-order CFAs revealed problems with misspecification for both classic and modern scales, establishing that newer does not necessarily imply better.

Finally, results revealed that at least one factor from each of the five scales examined here—both classic and modern—is driven by the same underlying attitude. As Heberlein 1981, p. 252) surmised almost 40 years ago, the second-order CFA does indeed suggest that a majority of these scales “all measure some general orientation.” Thus, although authors have often argued that the measurement of environmental concern has been scattered and divided (e.g., Dunlap and Jones, 2002), established scales are remarkably consistent in actuality.

### Recommendations and Future Directions

Two general recommendations are offered for scholars seeking to measure environmental concern. First, although the evidence suggests that all of the instruments analyzed here could be used to measure environmental attitudes, there would be a number of benefits of using the Schultz (2001) environmental concerns scale in future studies. In addition to producing excellent fit to the data, this scale had by far the highest reliabilities of any instrument. The Schultz scale also has the benefit of being one of the shortest instruments among those reviewed. In applied research, where survey length may be particularly important, this scale’s brevity is a clear advantage. Thus, this scale would be an excellent choice for any study of general environmental attitudes, bearing in mind that the *egoistic* and *social-altruistic concern* factors are distinct from environmental concern as represented by the *biospheric concern* factor.

Second, the second-order CFA findings can be interpreted as a cautionary tale for scholars interested in developing new scales. Specifically, these studies revealed that all five scales, at least in part, were measuring the same thing—general environmental concern or attitudes. In other words, most of the scales developed after Maloney and Ward’s (1973) first attempt have merely offered different ways of finding the same information. As such, we recommend that scholars interested in measuring general environmental concern use and work to improve existing instruments. Likewise, we recommend that applied environmental research make use of these scales, rather than developing *ad hoc* instruments for a specific study.

Additional measurement work will also help resolve why some of the factors investigated failed to load on the second-order unidimensional factor. For one, the second-order CFA revealed that EC scale’s *rejection of industrial status quo* factor and the LT scale’s *concern for overpopulation* factor are invalid indicators of environmental concern, but the reasons why these factors failed to load with the others are unclear. Future measurement studies can investigate this finding further by attempting to identify which underlying construct(s) drive these other factors, as well as by replicating the second-order model produced here, to ensure it is not an artifact of sampling error (Anderson and Gerbing, 1988).

Additional measurement work on these scales can also be beneficial in other ways. For example, the factors that were found to have low reliabilities, such as the LT scale’s *concern for environmental action* factor, can be augmented by additional valid items (Nunnally et al., 1967). Future authors can also attempt to replicate the factor structures produced herein by subjecting the full battery of items to similar CFAs (Anderson and Gerbing, 1988), and can conduct additional CFA work on the other scales presented in Table 1. Moreover, future authors can apply different methodological techniques that provide additional diagnostic information not provided in a CFA. Zhu and Lu (2017), for instance, use item-response theory to show that some items of the NEP are more reliable than others, especially when subjects’ attitudes become more extreme. Ultimately, when paired with similar tests of dimensionality like those reported here, such information would be valuable.

### Limitations

One important limitation of the present research is that the samples were obtained using mTurk, meaning that neither is nationally representative. Consequently, additional research with nationally representative samples is warranted if researchers wish to make claims regarding the generality of the measurement indices reported herein. Such research could speak to the fit of environmental concern scales in specific affiliation groups (e.g., general public vs. environmental organizations; Dunlap and Van Liere, 1978), in different countries (e.g., Xiao and Dunlap, 2007; Milfont and Duckitt, 2010), or in other segments that may be of interest to applied researchers. This type of work would also enable comparisons of structural validity across different groups and samples (Levine, 2005), as well as provide information about related forms of validity not discussed in this manuscript (e.g., predictive or criterion validity; Cronbach and Meehl, 1955). Moreover, such work could investigate further whether other environmental concern scales conform to the second-order factor produced in our data (for other likely candidates, see Table 1).

In addition, considering the broader debate about measurement approaches that are currently ongoing in this literature, the focus on traditional environmental attitude scales is also a limitation. In particular, Kaiser and colleagues (Kaiser et al., 2010, 2018; Kaiser and Wilson, 2019) have raised two major criticisms of traditional attitude scales. For one, they argue that attitude scales like the ones reviewed here are not effective predictors of behavior. Based on this premise, they advocate instead for an alternative measurement approach based on the Campbell paradigm, which argues that “the cost order of behavior…should be used as the basis for the measurement of individual attitudes” (Kaiser and Wilson, 2019, p. 362). In addition, Kaiser et al. (2018) argue that attitudes themselves may not be distinct from related constructs like subjective norms and behavioral intentions, given the strong correlations among them in many studies. These points raise broad questions about the predictive validity of attitude scales and the cognitive structure of attitudes that cannot explicitly be addressed with the results reported here.

There are several good reasons, however, not to abandon traditional approaches to attitudes. For one, meta-analyses demonstrate that attitude scales often do correlate strongly with intentions and behavior after accounting for methodological artifacts (Kim and Hunter, 1993a, b), which tempers the claim that they are not effective predictors of behavior. Furthermore, longitudinal studies have provided convincing evidence that attitudes, intentions, and behavior are causally related (Morrison et al., 1998; Vincent et al., 1998), which contradicts the claim that they are driven by a single underlying factor (see also the path analyses in Kim and Hunter, 1993b). There are also practical reasons to prefer scales with linear item-total correlations (like the ones employed here). For instance, the methodological paradigm adopted by Kaiser and colleagues requires items that are rank ordered (i.e. items that have ogival item-characteristic curves and thus conform to a Guttman simplex), which require more demanding methods and analyses to infer construct validity (see Keating and Boster, 2019; for other examples, see Kaiser et al., 2007; Arnold et al., 2018; Kaiser and Wilson, 2019). Especially for applied environmental researchers, who may have greater limitations on their time and resources, traditional attitude scales thus remain an appealing choice.

Nevertheless, we acknowledge that the debate between these approaches is still ongoing, and studies that make direct comparisons between the two (e.g., Brügger et al., 2011; Otto et al., 2018) can better inform this debate in the future. Additional measurement work can also examine the second-order dimensionality of not just attitudes, but intentions, norms, and other related constructs in a more rigorous fashion.

## Conclusion

Overall, this study has offered an important update to measurement work in the field of environmental concern, including a review of the available scales and an analysis of the structural validity of five prominent instruments that adhere to classical test theory. For applied scholars, we hope this information provides a helpful guide in navigating the literature on the measurement of environmental concern and attitudes. By using the established scales recommended here, scholars can be confident they are using valid and reliable instruments, and hopefully avoid some of the measurement problems that have plagued other authors when studying this topic in the past.

## Data Availability Statement

The datasets generated for this study are available on request to the corresponding author.

## Ethics Statement

The studies involving human participants were reviewed and approved by Institutional Review Board – Michigan State University. Written informed consent for participation was not required for this study in accordance with the national legislation and the institutional requirements.

## Author Contributions

SC wrote substantial portions of the manuscript, and both collected and analyzed data. BM performed the same tasks.

## Conflict of Interest

The authors declare that the research was conducted in the absence of any commercial or financial relationships that could be construed as a potential conflict of interest.

## Footnotes

1 CFI and AIC values were obtained using maximum likelihood estimation.

2 Although some authors argue that dropping items from a scale makes the analysis exploratory rather than confirmatory (e.g., Browne, 2001; Harrington, 2008), we, like other authors (e.g., Levine et al., 2006), disagree. Rather than reifying specific items as necessary components of a scale, we view them as random samples from an infinite universe of possible items (Kerlinger, 1964). To wit, it makes little sense to present an invalid statistical model that includes these items when a valid one can be attained without them. Thus, in using CFA we use *confirmatory* to refer to a specific statistical model used to test a specified factor structure, and not to refer to a rigid requirement that no changes be made.

3 To reiterate, this means that observed correlations deviated substantially from the correlations predicted by the internal consistency theorem. It does not refer to reliability, which is sometimes also referred to as internal consistency.

4 This scale was also designed with a 15-item knowledge subscale, but this subscale was excluded from the analysis because (1) it was presumably non-linear; and (2) because the items would need to be scored incorrect/correct, rather than on a five-point scale comparable to the other measures.

5 When the actual commitment items were originally written, they were designed as true/false items. In order to capture these items on five-point scales, some of the items needed to be reworded. For example, the item “I guess I’ve never actually bought a product because it had a lower polluting effect [T/F]” was reworded as “I buy products because they have a lower polluting effect [never – regularly].”
